# Focal adhesion kinase activity is required for actomyosin contractility-based invasion of cells into dense 3D matrices

**DOI:** 10.1038/srep42780

**Published:** 2017-02-16

**Authors:** Claudia T. Mierke, Tony Fischer, Stefanie Puder, Tom Kunschmann, Birga Soetje, Wolfgang H. Ziegler

**Affiliations:** 1 Institute of Experimental Physics I, Biological Physics Division, Faculty of Physics and Earth Science, University of Leipzig, Leipzig, Germany; 2 Department of Paediatric Kidney, Liver and Metabolic Diseases, Hannover Medical School, Hannover, Germany

## Abstract

The focal adhesion kinase (FAK) regulates the dynamics of integrin-based cell adhesions important for motility. FAK’s activity regulation is involved in stress-sensing and focal-adhesion turnover. The effect of FAK on 3D migration and cellular mechanics is unclear. We analyzed FAK knock-out mouse embryonic fibroblasts and cells expressing a kinase-dead FAK mutant, R454-FAK, in comparison to FAK wild-type cells. FAK knock-out and FAK^R454/R454^ cells invade dense 3D matrices less efficiently. These results are supported by FAK knock-down in wild-type fibroblasts and MDA-MB-231 human breast cancer cells showing reduced invasiveness. Pharmacological interventions indicate that in 3D matrices, cells deficient in FAK or kinase-activity behave similarly to wild-type cells treated with inhibitors of Src-activity or actomyosin-contractility. Using magnetic tweezers experiments, FAK^R454/R454^ cells are shown to be softer and exhibit impaired adhesion to fibronectin and collagen, which is consistent with their reduced 3D invasiveness. In line with this, FAK^R454/R454^ cells cannot contract the matrix in contrast to FAK wild-type cells. Finally, our findings demonstrate that active FAK facilitates 3D matrix invasion through increased cellular stiffness and transmission of actomyosin-dependent contractile force in dense 3D extracellular matrices.

Cell adhesion is a process that regulates the interaction of cytoskeletal filaments with the local microenvironment and thus is necessary for the regulation of tissue homeostasis and tissue repair after injury^1^. The adhesion process also plays a fundamental role in cancer progression and metastasis^2^,^3^,^4^. Cell-surface expressed integrins connect the extracellular matrix to cytoskeletal microfilaments. This connection initiates signaling to the cell by clustering a complex of proteins collectively termed focal adhesions^5^,^6^,^7^ and recently multimolecular integrin adhesion complex^8^,^9^. Focal adhesion proteins such as vinculin and focal adhesion kinase (FAK) are critical for the process of cell invasion in extracellular matrices^10^,^11^,^12^,^13^,^14^.

FAK is a cytoplasmic non-receptor tyrosine kinase, which associates closely with integrins and, when activated localizes to cell-matrix contact sites, the focal adhesions^15^,^16^,^17^. The activation of FAK is characterized by autophosphorylation at Tyr-397, providing in its phosphorylated state a docking site for Src, which leads to further FAK phosphorylation at Tyr-576 and Tyr-577 by Src, maximal adhesion-induced FAK activation and the assembly of a large signaling complex^18^,^19^,^20^,^21^. At focal adhesions, FAK has two main functions; firstly, as a cytoskeleton-associated scaffolding protein and secondly, as a kinase-mediating integrin-dependent tyrosine phosphorylation^22^. The kinase activity of FAK leads to signaling via PI3K/Akt and MAPK pathways and inhibits apoptosis^16^. Expression of dominant-negative FAK mutant constructs evokes enhanced apoptosis associated with decreased cell adhesion and subsequently, decreased adhesion-facilitated cell survival^23^,^24^.

By contrast, overexpression of FAK suppresses apoptosis through the nuclear factor kappa B (NF-kB) pathway^25^. FAK promotes survival by facilitating ubiquitin-based degradation of the tumor suppressor protein p53. Under cellular stress induced by DNA damage, hypoxia or oncogene activation, FAK translocates into the cell nucleus mediating p53 degradation and subsequently, cell survival^26^,^27^,^28^.

In addition, FAK functions in cellular mechanics as its activity depends on the rigidity of the microenvironment and it is supposed to be (part of) a mechanosensor of tissue rigidity^29^,^30^. Further, FAK promotes proliferation in response to decreased tissue compliance through upregulation of cyclin D^31^. Within cells, focal adhesion, or stretch-activated signaling pathways, as well as myosin II appear to act as mechanosensors. They operate by transducing signals to downstream regulatory proteins in response to the mechanical properties of the microenvironment, and, by the induction of force-dependent stress-stiffening of the cells as detected by magnetic twisting cytometry^17^,^32^. In addition, low substratum rigidity induces down-regulation of focal adhesion proteins such as FAK, indicating a mechano-response behavior, which is a critical aspect of the regulation of cellular motility^33^.

FAK regulates the assembly and disassembly of focal adhesions which are necessary for cell motility. Overexpression of FAK increases cell motility^34^, whereas FAK-deficient cells and overexpression of a dominant-negative FAK (FRNK = FAK-related non kinase) show increased focal adhesion numbers and hence decreased motility^35^,^36^. In line with this, transfection of wild-type FAK cDNA into FAK-deficient cells restores cell migration, but not transfection of the Y397F FAK mutant cDNA, encoding an FAK mutant deficient in kinase signaling^37^. Besides FAK, there are several other signal pathways which regulate cell migration such as mitogen-activated protein kinases (MAPK), Jun N-terminus kinase (JNK) and p38, which all play a role in cell invasion^38^. Among these, the activation of ERK1/2 is most important for cell spreading and migration^39^,^40^,^41^,^42^,^43^.

It is widely accepted that FAK located in focal adhesions plays a role in regulating the initiation of cellular motility^14^,^16^,^26^,^44^, however also other focal adhesions proteins play a role in cellular migration^12^,^43^. The aim of our study was (i) to investigate the role of FAK in cancer cell and fibroblast invasion under controlled *in-vitro* conditions, and (ii) to define the cell mechanical aspects of invasion, which depend on FAK kinase activity. We used 2.4 mg/ml artificial 3D extracellular matrix protein matrices with subcellular-sized pores for invasion assays^45^,^46^,^47^. Using this approach, the invasiveness of cells depends on mechanical processes including cell adhesion and de-adhesion^48^, cytoskeletal remodeling^47^, generation of protrusive forces^48^,^49^, and matrix properties such as rigidity, pore size, extracellular matrix protein composition as well as proteolytic degradation^50^.

In this study, we examined whether expression of FAK facilitates 3D extracellular matrix invasion through enhanced cellular stiffness and increases cell adhesion to fibronectin, as required to overcome steric restriction of dense 3D extracellular matrices. FAK wild-type mouse embryonic fibroblasts display increased invasiveness into 3D extracellular matrices compared to FAK knock-out cells. In line with this, knock-down of FAK (and Pyk2) in FAK wild-type fibroblasts decreased cell invasion into 3D extracellular matrices. We determined the characteristics of the FAK invasion-enhancing effect by analyzing cell adhesion strength and cellular stiffness. In addition, we explored whether the FAK-facilitated invasiveness depends on Erk and Src signaling and by using specific inhibitors, we confirmed their impact on invasiveness. Furthermore, by inhibiting ROCK and MLCK, we observed in wild-type fibroblasts that FAK activity facilitates invasiveness in 3D extracellular matrices dependent mostly on contractile forces, whereas the low invasiveness of FAK^R454/R454^ cells is not sensitive to further inhibition and these cells exert impaired forces as reflected by comparably limited flow fields around these cells in 3D extracellular matrices. The residual invasiveness of FAK^R454/R454^ cells may depend on another migration mechanism based on actin polymerization-facilitated cell gliding. In summary, we found that FAK activity contributes substantially to the invasiveness of fibroblasts by providing cellular signaling, which coordinates the transmission of contractile and protrusive (compressive) forces towards extracellular matrix.

## Results

### FAK knock-out and FAK knock-down decrease cell invasiveness in 3D extracellular matrices

**Figure 1:**
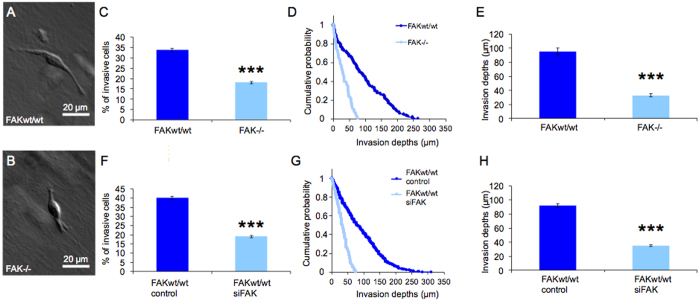
FAK expression correlates with increased invasiveness of fibroblasts into 3D extracellular matrices. (**A,B**) Representative images of invading cells showing FAK^wt/wt^ fibroblast (**A**) at 110 μm invasion depth and FAK^−/−^ fibroblast (**B**) at 43 μm of invasion depth. (**C**) Three days after seeding on dense 3D matrices, the relative number of invasive FAK^wt/wt^ cells, given as mean ± SD, is increased compared to FAK^−/−^ cells. (**D**) The invasion profiles and (**E**) the average invasion depths demonstrate a significantly higher invasiveness of FAK^wt/wt^ compared to FAK^−/−^ cells. Similar results were obtained by siRNA based knock-down of FAK in FAK^wt/wt^ cells using specific siFAK. (**F**) The percentage (mean ± SD) of invasive FAK^wt/wt^ cells treated with siFAK is markedly decreased compared to controls treated with unspecific control siRNA. (**G**) Invasion profile and (**H**) average invasion of FAK^wt/wt^ cells treated with siFAK confirm loss of invasive behavior after FAK knock-down. (n = 4, p*** < 0.001). Reduction of FAK to below 1/3 of control was verified by quantification of mRNA and protein levels (Supplementary Fig. S1 and Table S1).

To examine the effect of FAK protein on cell invasion, FAK knock-out (FAK^−/−^) and FAK wild-type (FAK^wt/wt^) mouse embryonic fibroblasts were analyzed. The invasiveness of fibroblasts can be determined by studying cell migration in *in-vitro* 3D extracellular matrices^12^. The invasiveness observed over a period of three days is characterized by the percentage of invasive fibroblasts and the invasion profile, which is expressed as the cumulative probability of invasive fibroblasts as a function of invasion depth. Representative micrographs of FAK^wt/wt^ and FAK^−/−^ fibroblasts are shown (Fig. 1A and B). The percentage of fibroblasts able to invade a 3D collagen matrix is higher in wild-type compared to knock-out cells (Fig. 1C). Moreover, the invasion profiles (cumulative probability) of the invading cells reveal that FAK^wt/wt^ cells invaded deeper into the 3D extracellular matrix (Fig. 1D and E). The invasion profiles of FAK^−/−^ cells show that these cells invade less than 100 μm (Fig. 1D) and the average invasion depth was reduced from 95.3 ± 4.8 μm of FAK^wt/wt^ cells (n = 212) to 33.1 ± 2.2 μm of FAK^−/−^ cells (n = 101) (Fig. 1E). After two days of specific siRNA treatment using siFAK, FAK expression in wild-type fibroblasts was reduced below 20% on protein level (Supplementary Fig. S1 and Table S1). FAK silencing decreased the number of invasive cells in 3D extracellular matrices significantly in contrast to control siRNA-treated cells (Fig. 1F). The invasion profiles of FAK knock-down fibroblasts show that these cells, similar to FAK knock-out cells, invade to less than 100 μm (Fig. 1G) and their average invasion depth was reduced from 92.0 ± 2.8 μm in controls (n = 526) to 35.3 ± 1.4 μm of siFAK-treated FAK^wt/wt^ cells (n = 196) (Fig. 1H). These results indicate that FAK protein expression correlates with increased fibroblast invasion in dense 3D collagen matrices.

### Pyk2 knock-down affects the invasiveness of FAK^wt/wt^ and not of FAK^−/−^ fibroblasts

**Figure 2:**
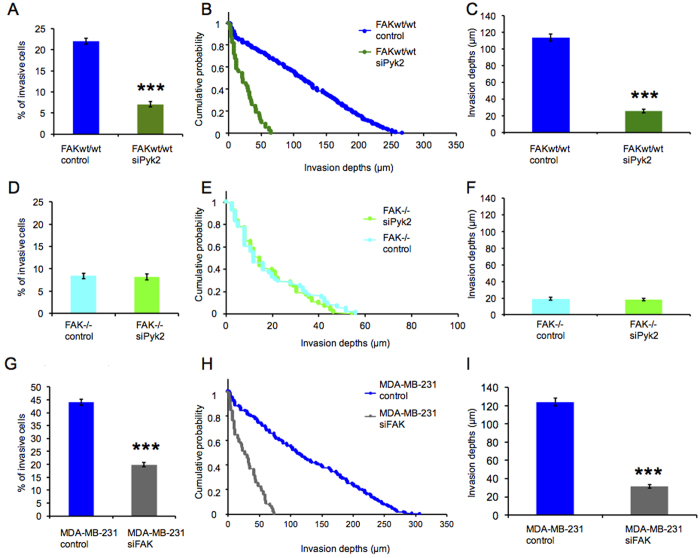
Reduction of Pyk2, in addition to FAK, can further reduce invasiveness of FAK^wt/wt^ cells. (**A**) After siPyk2 silencing, which efficiently suppresses FAK and Pyk2 expression (Supplementary Fig. S1 and Table S1), the percentage (mean ± SD) of invasive FAK^wt/wt^ cells treated with siPyk2 is significantly reduced compared to cells treated with unspecific control siRNA. (**B**) The invasion profile and (**C**) the average invasion depth of FAK^wt/wt^ cells treated with siPyk2 are greatly reduced compared to control and also to siFAK treatment affecting FAK protein only (Fig. 1F–H). By contrast, siPyk2 was ineffective in FAK^−/−^ cells and could not reduce significantly Pyk2 levels, at least compared to FAK^wt/wt^ cells (Supplementary Fig. S1 and Table S1). Consistently, (**D**) the percentage (mean ± SD) of invasive FAK^−/−^ cells, (**E**) the invasion profile and (**F**) the average invasion depth do not indicate any differences in FAK^−/−^ cells treated with siPyk2 in relation to respective controls. (n = 3, p*** < 0.001). (**G–I**) Effect of FAK expression on cancer cell invasiveness. Human MDA-MB-231 breast cancer cells were transfected with human-specific siFAK and control siRNA (Supplementary Fig. S1 and Table S1) to analyze the consequences of FAK knock-down. The invasiveness of siFAK-treated breast cancer cells is significantly reduced, characterized by the decreased percentage (mean ± SD) of invasive cells (**G**), the invasion profile (**H**) and the decrease in average invasion depths (**I**). (n = 3, p*** < 0.001).

A previous study reported that FAK knock-out cells show increased expression and phosphorylation of proline-rich tyrosine kinase 2 (Pyk2)^51^. In order to investigate a potentially compensating contribution of Pyk2 to the invasiveness of fibroblasts, we silenced Pyk2 in addition to FAK in FAK^wt/wt^ cells by use of a siRNA specific to FAK and Pyk2 (siPyk2). Quantitative mRNA analysis revealed a transient increase of Pyk2 expression, when only FAK was silenced, while siPyk2-mediated knock-down in FAK^wt/wt^ cells efficiently reduced both transcripts (Supplementary Table S1). As expected, siPyk2-silenced FAK^wt/wt^ cells, deficient for FAK and Pyk2 protein, showed a decrease in the percentage of invasive cells (Fig. 2A), an altered invasion profile (Fig. 2B) and also a reduction in the mean invasion depths from 113.5 ± 4.6 μm in controls (n = 273) to 25.3 ± 2.2 μm of siPyk2-treated FAK^wt/wt^ cells (n = 74) (Fig. 2C). Both, number of invasive cells and invasion depth were further reduced compared to siFAK-treated FAK^wt/wt^ cells (Fig. 1F,H) confirming that Pyk2 contributes to matrix invasion and can partially compensate acute loss of FAK. When silencing FAK^−/−^ cells using siPyk2, no (additional) effect on the number of invasive cells, invasion probability or depth was observed compared to control-treated cells (Fig. 2D–F). The invasion profiles of FAK^−/−^ cells with or without knock-down of Pyk2 show minimal matrix invasion, low probabilities and invasion depths far below 100 μm, with an average invasion depth of 18.5 ± 1.5 μm (n = 88) for control siRNA-treated cells (Fig. 2E) and 19.1 ± 1.7 μm (n = 82) for specific siPyk2-treated cells (Fig. 2F), respectively. Quantitative mRNA analysis revealed that siPyk2 treatment was not effective in FAK^−/−^ cells. Although Pyk2 expression was moderately reduced, compared to FAK^wt/wt^ cells, siPyk2-treated FAK^−/−^ cells maintained 3–4 fold enhanced Pyk2 expression (Supplementary Table S1). Our results indicate that Pyk2 contributes to the invasive behavior of cells as part of the FAK signaling pathway. In FAK^−/−^ cells, which are deficient in FAK protein and thus signaling, Pyk2 does not appear to sustain the invasive behavior of cells and cannot compensate FAK function substantially, with respect to cell invasion in dense 3D extracellular matrices.

### Invasiveness of human breast cancer cells also depends on FAK expression

In order to address the general relevance of FAK expression on cell invasiveness, FAK silencing was tested in the human breast cancer cell line MDA-MB-231. Cancer cells were treated with control siRNA and human FAK-specific siRNA (Supplementary Fig. S1 and Table S1). On studying siRNA-treated cells in the 3D matrix invasion assay, we found that after FAK knock-down, human MDA-MB-231 breast cancer cells displayed a decreased amount of invasive cells (Fig. 2G), an invasion profile shifted towards decreased invasion probability (Fig. 2H) and a reduction in average invasion depth (Fig. 2I). The average invasion depth of 123.9 ± 4.3 μm (n = 381) for cells treated with control siRNA was reduced to 31.5 ± 2.2 μm (n = 105) after treatment of MDA-MB-231 cells with FAK-specific siRNA (Fig. 2I). Changes are comparable to those observed in FAK knock-down fibroblasts, demonstrating that the effect of FAK on cellular invasiveness is not restricted to a particular cell type, i.e. mouse embryonic fibroblasts. Furthermore, a recent study using MDA-MB-231 cells demonstrated that inhibition of FAK’s kinase activity leads to reduced motility and migration speed in 3D collagen microtracks^52^. These observations in human breast cancer cells suggest that FAK can play a functional role in supporting cancer cell invasiveness and, finally, promote metastasis formation.

### FAK^R454/R454^ cells exhibit impaired cell invasion

**Figure 3:**
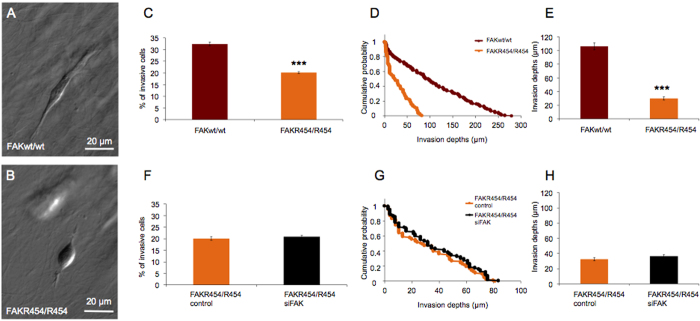
Impact of FAK kinase activity on matrix invasion of fibroblasts. Invasiveness of hTERT-immortalized FAK^wt/wt^ and FAK^R454/R454^ cells was analyzed in the 3D extracellular matrix invasion assay. (**A,B**) Representative images of invading cells showing FAK^wt/wt^ cells (**A**) at 122 μm invasion depth and FAK^R454/R454^ cells (**B**) at 39 μm invasion depth. (**C**) The percentage (mean ± SD) of invasive cells is significantly higher for FAK^wt/wt^ compared to FAK^R454/R454^ cells. (**D**) Invasion profiles and (**E**) invasion depths are also altered. Average invasion depths of FAK^wt/wt^ cells is significantly higher than that of FAK^R454/R454^ cells. (**F–H**) Effects of siRNA-mediated FAK-silencing (Supplementary Fig. S1 and Table S1) on invasiveness of FAK^R454/R454^ cells were investigated. Compared to controls, siFAK treatment does neither alter (**F**) the percentage (mean ± SD) of invasive FAK^R454/R454^ cells, nor (**G**) the invasion profiles or (**H**) the average invasion depths of cells. (n = 4, p*** < 0.001).

To analyze whether an active FAK kinase domain is required for cell invasion, we investigated a cell line expressing the ATP-binding deficient Lys454-to-Arg mutation of FAK (R454 FAK), which lacks kinase activity^11^, and FAK wild-type control cells in the 3D extracellular matrix invasion assay. These cell lines, provided by Dr. David D. Schlaepfer, were derived from E8.5 mouse embryos and preserved using retrovirus-facilitated expression of human telomerase reverse transcriptase (hTERT)^11^. Wild-type and R454 FAK mutant expressing cells showed corresponding differences in their morphologies inside the collagen matrices (Fig. 3A and B) as was observed for control and FAK^−/−^ cells (Fig. 1A and B). Further, we observed that FAK^R454/R454^ cells are less invasive than FAK wild-type cells as determined by a decreased number of cells which invaded the 3D extracellular matrices (Fig. 3C), as well as a reduced probability and decreased depth of invasion (Fig. 3D and E). The average invasion depth was 30.0 ± 2.3 μm (n = 117) and 106.1 ± 5.2 μm (n = 233) of FAK^R454/R454^ and FAK wild-type cells, respectively (Fig. 3E). In addition, knock-down of the R454 FAK mutant protein using FAK-specific siRNA (siFAK; Supplementary Fig. S1 and Table S1) did not lead to significant differences in invasiveness, either in the numbers of invasive cells (Fig. 3F) nor in their invasion depths (Fig. 3G and H). For control siRNA and siFAK treated FAK^R454/R454^ cells, nearly identical average invasion depths were observed with 32.5 ± 2.3 μm (n = 118) and 36.6 ± 2.3 μm (n = 122), respectively (Fig. 3H). These results reveal that FAK kinase activity is a critical requirement for FAK function supporting cell invasiveness, whereas removal of the R454 FAK mutant protein, i.e. protein scaffold function without Tyr-397 phosphorylation, does not further reduce the invasive behavior of FAK^R454/R454^ cells lacking the signaling function of FAK kinase. As a previous report showed that defects in cell adhesion may cause decreased extracellular matrix remodeling^53^, we investigated the effect of this by inhibiting matrix degradation. In particular, the effect of FAK on collagen degradation by matrix-metalloproteinases was investigated using the broad-spectrum matrix-metalloproteinase inhibitor GM6001^45^ (50 μM). Invasiveness of the FAK ^R454/R454^ and FAK wild-type cells was significantly different between both cell types independent of treatment, and not significantly altered for each cell type in the presence of GM6001 as compared to vehicle treatment (Supplementary Fig. S2). These results suggest that matrix degradation is not a relevant alternative to the invasion strategy of both cell types under these 3D matrix constraints, however, it may play a role when sterically more restrictive, i.e. cross-linked, matrices are used for migration analysis.

### FAK kinase activity is required for cellular control of stiffness and adhesion strength

**Figure 4:**
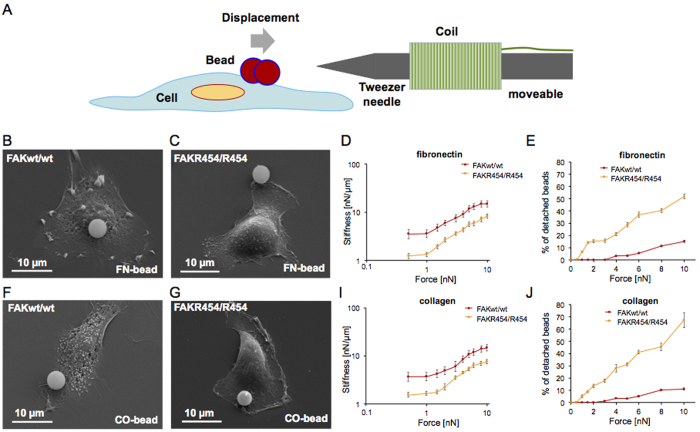
Stiffness and adhesion strength are reduced substantially in FAK^R454/R454^ as compared to FAK^wt/wt^ cells. (**A**) Scheme of magnetic tweezers setup. Representative scanning electron microscopic images of FAK^wt/wt^ and FAK^R454/R454^ cells, binding beads coated with fibronectin (**B** and **C**, respectively) or collagen type I (**F** and **G**, respectively). Prior to application of defined forces through magnetic tweezers, beads were bound to cells for at least 30 min. Measurements with fibronectin- (**D**) and collagen-coated beads (**I**) show decreased stiffness of FAK^R454/R454^ cells (orange) compared to FAK^wt/wt^ cells (red). Insufficient adhesion strengthening of FAK^R454/R454^ cells is revealed by high incidence of detachment/loss of both fibronectin- (**E**) and collagen-coated beads (**J**) at low forces, which was not observed for wild-type FAK protein expressing control cells. Values of stiffness and adhesion strength are expressed as mean ± SD (n = 3, > 140 cells for each condition).

In order to investigate whether stiffness and adhesion strength, both mechanical properties essential for cell invasion, are being modulated by FAK kinase activity, we analyzed FAK^wt/wt^ cells and FAK^R454/R454^ cells with magnetic tweezers to determine both parameters. In particular, we applied forces of up to 10 nN to superparamagnetic beads, which were coated with fibronectin (an extracellular matrix protein) or collagen type I and bound to the cells. The fibronectin or collagen on the beads connects integrins, which are themselves tethered to contractile actin filaments through proteins in the focal adhesion complex^12^. The experimental setup is illustrated in Fig. 4A. Scanning electron microscopic images show fibronectin-coated beads bound to FAK^wt/wt^ (Fig. 4B) and FAK^R454/R454^ cells (Fig. 4C). During a step-wise increase in force (creep measurement), bead displacement follows a power-law^12^,^47^. Stiffness measurements revealed significantly higher mean cell stiffness when cells expressed FAK wild-type protein compared to the kinase-dead R454 FAK mutant, (Fig. 4D). These results indicate that in focal adhesions, expression of FAK wild-type protein, whose kinase activity is induced upon engagement of integrins, increases cellular stiffness and can thus facilitate cell migration into 3D extracellular matrices. Moreover, the reduced stiffening response observed in FAK^R454/R454^ cells, suggests that transmission of contractile forces appears to be important in FAK facilitated cell invasion. Similar results were obtained in experiments using collagen-coated beads. Bead binding to FAK^wt/wt^ and FAK^R454/R454^ cells is shown in scanning electron microscopic images (Fig. 4F and G, respectively). In FAK^wt/wt^ cells, cellular stiffness is higher than in FAK^R454/R454^ cells (Fig. 4I) and differences in profiles are comparable to those observed for fibronectin-coated beads. These results indicate that cellular stiffness and stiffening response are increased in FAK^wt/wt^ cells independent of the bead coating. Consistently, high incidence of bead detachment reveals a highly decreased strength of adhesion of fibronectin-coated and collagen-coated beads to FAK^R454/R454^ compared to FAK^wt/wt^ cells (Fig. 4E and J, respectively). This process is entirely integrin-dependent, as the addition of a blocking antibody for β1 integrin abolishes completely the binding of fibronectin-coated beads to fibroblasts^45^.

**Figure 5:**
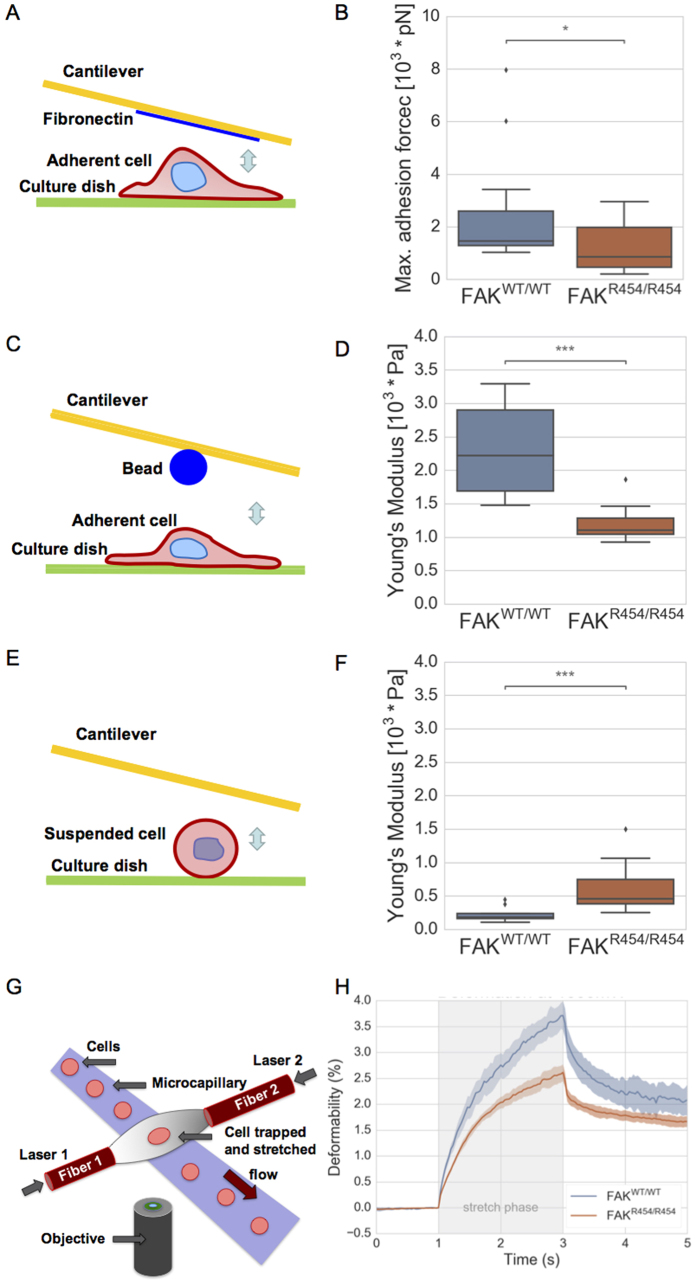
Comparing FAK^wt/wt^ to FAK^R454/R454^ cells, atomic force microscopy determines higher cell-matrix adhesion forces and cellular stiffness (Young’s modulus) for adherent FAK^wt/wt^ cells. (**A**) Scheme of setup used for measurement the cell-matrix adhesion force measurements using atomic force microscopy with a flat fibronectin-coated cantilever. (**B**) The maximum adhesion forces of FAK^wt/wt^ cells (blue, n = 23) are higher compared to FAK^R454/R454^ cells (red, n = 19). (**C**) Schematic drawing of the atomic force microscopic setup used for the stiffness measurement of adherent cells with a bead glued to a cantilever. (**D**) The Young’s modulus of adherent FAK^wt/wt^ cells (blue, n = 23) is higher compared to adherent FAK^R454/R454^ cells (red, n = 19). (**E**) Schematic drawing of the atomic force microscopic setup used for the stiffness measurement of suspended cells with a flat cantilever. (**F**) The Young’s modulus of suspended FAK^wt/wt^ cells (blue, n = 20) is higher compared to suspended FAK^R454/R454^ cells (red, n = 20). (**G**) Schematic drawing of the optical cell stretcher setup used for deformability measurements of suspended cells. (**H**) The deformability of suspended FAK^wt/wt^ cells using an optical cell stretcher (blue, n = 251) is increased compared to suspended FAK^R454/R454^ cells (red, n = 710), at a laser stretch power of 1000 mW. (p*/p*** < 0.05/0.001).

To further analyze cellular stiffness in relation to cell adhesion, we performed atomic force microscopic measurements of adherent and suspended FAK^wt/wt^ and FAK^R454/R454^ cells (Fig. 5). We found that adherent FAK^wt/wt^ cells possess a higher adhesion strength compared to FAK^R454/R454^ cells (Fig. 5A and B). By using the scanning mode of the atomic force microscope with a cantilever to which a bead was glued, we determined the stiffness of cells measuring the Youngs’ modulus (Fig. 5C and D). FAK^wt/wt^ cells have a higher Youngs’ modulus than FAK^R454/R454^ cells corresponding to a higher cell stiffness. To determine the Young’s modulus without the influence of integrin-dependent adhesion, we measured non-adherent cells using the scanning mode of the AFM and a flat non-coated cantilever (Fig. 5E and F). As anticipated, in the absence of substrate adhesion, cellular stiffness (Young’s modulus) is reduced strongly in both cell types, FAK^wt/wt^ and FAK^R454/R454^ cells (Fig. 5F). However, the preserved cellular stiffness observed is significantly higher in FAK^R454/R454^ cells than in FAK^wt/wt^ cells (Fig. 5F). To further verify stiffness of the cells without adhesion, cells were analyzed using an optical cell stretcher device illustrated in Fig. 5G. Deformation of the cells along the axis parallel to the laser beams shows that the FAK^wt/wt^ cells are more deformable, which means the non-adherent FAK^R454/R454^ cells are stiffer (Fig. 5H). Together, these results indicate that matrix adhesion is central to the overall cellular stiffness and hence the tensional state of both cell types. In FAK^R454/R454^ cells, cellular mechano-coupling is reduced and thus the induction of stiffness upon adhesion is impaired compared to FAK^wt/wt^ cells.

### Control of cell morphology and matrix remodeling are affected by FAK kinase activity

**Figure 6:**
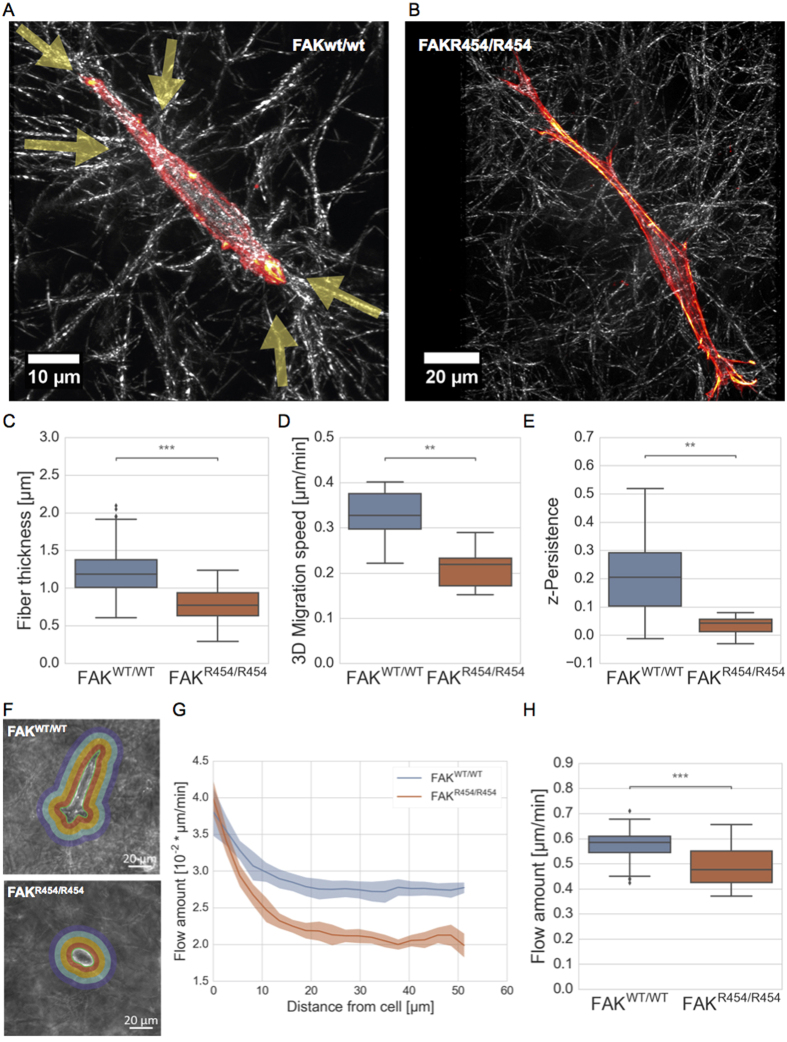
Cell morphology and actin cytoskeleton of fibroblasts remodeling collagen matrix. Representative confocal laser-scanning micrographs show (**A**) FAK^wt/wt^ cell and (**B**) FAK^R454/454^ cell migration through dense 3D collagen matrix (2.4 mg/ml). Overlay images reveal actin cytoskeleton/stress fibers (AlexaFluor546-Phalloidin, red) of cells embedded in a 3D collagen fiber network detected by reflection mode imaging. Arrows highlight areas of matrix contraction, which were observed only for FAK^wt/wt^ cells (n = 3, at least 13 to 25 invasive cells were analyzed). (**C**) The stress fiber thickness of FAK^wt/wt^ cells is significantly bigger compared to FAK^R454/R454^ cells, in addition, (**D**) migration speed and (**E**) persistence of invasion in 3D collagen matrices are increased significantly in FAK^wt/wt^ cells. (**F**) Images illustrating the flow field around one representative FAK^wt/wt^ cell (top) and FAK^R454/R454^ cell (bottom), each. The flow fields of cell migration in 3D collagen matrix for both cell types is shown as (**G**) the radial distribution of flow around the cells and (**H**) the total amount of flow for 1 hour over all length scales. (n = 3, p**/p*** < 0.01/0.001).

In order to monitor differences in actin cytoskeletal organization and matrix remodeling of FAK wild-type expressing fibroblasts in relation to cells expressing the R454 FAK mutant, both cell lines were seeded and cultured on 3D extracellular matrices. After two days, cells were fixed and stained with AlexaFluor546 phalloidin. Using laser scanning confocal microscopy, the actin cytoskeleton and cellular morphology were analyzed by taking z-stacks (n = 21 for FAK^wt/wt^ and n = 49 for FAK^R454/R454^ in at least three repeat experiments for each cell type, the pitch for both was 0.33 μm) of fluorescence and reflection mode images to reveal (i) actin fiber networks, and, (ii) the cells within the 3D collagen matrix, respectively. While migrating through dense matrices, both cell types assemble actin stress fibers, acquire a fibroblast-like morphology and align themselves along collagen fibrils (Fig. 6A and B). The median thickness of stress fiber determined for invaded FAK^wt/wt^ cells is significantly bigger than observed in FAK^R454/R454^ cells (Fig. 6C). In addition, FAK^wt/wt^ cells possess a significantly higher speed (Fig. 6D) and persistence (directionality) of migration (Fig. 6E) compared to FAK^R454/R454^ cells, as determined from time-lapsed video images of cells migrating in 3D extracellular matrices. While FAK^wt/wt^ cells typically contract the collagen fiber network, as highlighted by arrows (Fig. 6A), FAK ^R454/R454^ cells failed to detectably alter the matrix (Fig. 6B). Furthermore, videos of invading cells show that FAK^wt/wt^ cells deform their surrounding 3D matrix stronger than FAK^R454/R454^ cells (Supplementary videos S1 and S2, showing one representative cell for FAK^wt/wt^ and FAK^R454/R454^, respectively). In addition, we analyzed the amount of flow around invading FAK^wt/wt^ cells (Fig. 6G top) and FAK^R454/R454^ cells in certain distances (Fig. 6G bottom). Representative images of both cell types show different morphology, polarity or cell volume (and area) alterations due to their invasion depths (Fig. 6F). Compared to FAK^R454/R454^ cells, the flow fields around FAK^wt/wt^ cells are significantly enhanced at different length scales around invading cells, as analyzed over at least 1.5 to 3 hours, (Fig. 6G) and the total flow values of FAK^wt/wt^ cells over all length scales are also significantly higher (Fig. 6H). This suggests that fibroblasts expressing a kinase-dead version of FAK only, are defective in integrin-dependent matrix remodeling and/or transmission of forces, which may explain their inability to invade dense 3D extracellular matrices efficiently as observed for FAK wild-type expressing control cells.

### FAK^R454/R454^ cells lack Erk and Src signaling- mediated invasiveness

**Figure 7:**
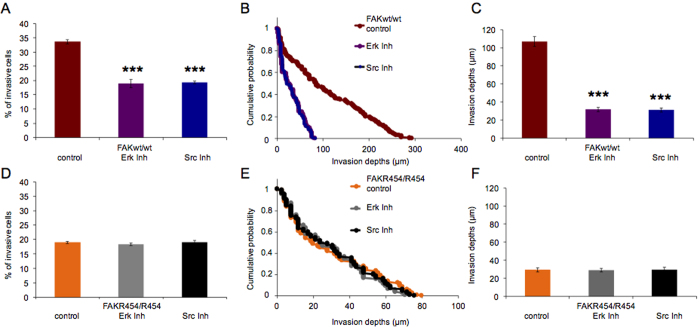
Inhibition of Erk or Src-kinase signaling strongly affects invasiveness of FAK^wt/wt^ cells and does not further reduce the invasiveness of FAK^R454/R454^ cells. Reduced invasiveness of FAK^wt/wt^ cells after Erk and Src inhibitor-treatment, compared to vehicle treatment, is characterized (**A**) by a significantly reduced percentage (mean ± SD) of invasive cells and by decreased invasion depths as indicated (**B**) by reduced invasion profiles and (**C**) by significantly lower mean invasion depths. In FAK^R454/R454^ cells, Erk and Src inhibitors have no effect on cell invasiveness. (**D**) The percentage of invasive cells is not altered, (**E**) invasion profiles are identical, and (**F**) the average invasion depths are also unchanged. (n = 4, p*** < 0.001).

Normally, FAK-facilitated cellular invasiveness depends on Erk and Src kinase signaling^54^. To quantify the impact of these kinases, we performed the 3D extracellular matrix invasion assay to study FAK^wt/wt^ cells in the presence and absence of the Erk inhibitor (Erk inh., 20 μM; Calbiochem) and the Src inhibitor (Src inh., 30 μM; Calbiochem), respectively. Both inhibitors markedly decreased the invasiveness of FAK^wt/wt^ cells compared to vehicle treatment as determined by reductions in the percentages of invasive cells (Fig. 7A), altered invasion profiles and the reduction in the invasion depths of these cells (Fig. 7B and C). The average invasion depths dropped by two thirds from 106.7 ± 5.5 μm (n = 247) observed for vehicle-treated controls to 31.8 ± 2.3 μm (n = 113) for Erk inh. and 31.1 ± 2.2 μm (n = 119) for Src inh.-treated cells, respectively (Fig. 7A–C). These results indicate that migration of FAK wild-type expressing cells into 3D extracellular matrices depends strongly on Erk and Src kinase-signaling. In adhesion sites, lack of FAK kinase activity, which is known to modulate Erk and Src signaling in cells^16^,^49^, appears to be a prime cause of defective invasiveness seen for FAK^R454/R454^ cells. Analyses of cell invasiveness using cells that express the kinase-dead R454 FAK mutant protein only, consistently do not show any change in the presence of inhibitors for Erk or Src kinase activity compared to vehicle treatment. We found that both inhibitors did not alter the number of invasive FAK^R454/R454^ cells (Fig. 7D), their invasion depths indicated by the invasion profiles (Fig. 7E), or their invasion depths (Fig. 7F). Notably, the average invasion depths were identical to 29.5 ± 2.2 μm (n = 115), determined for vehicle-treated controls, and 29.2 ± 1.9 μm (n = 107) for Erk inh. and 29.8 ± 2.1 μm (n = 112) for Src inh.-treated cells, respectively (Fig. 7A–C). These results suggest that a functional, active kinase domain of FAK plays a pivotal role in forming strong adhesions, supporting adhesion-dependent signaling and finally promoting cell migration into 3D extracellular matrices. Thus, the functional FAK protein facilitates invasiveness of cells based on pathways that depend on Erk and Src kinase- signaling.

### FAK kinase activity is required for actomyosin contractility-mediated cell invasion

**Figure 8:**
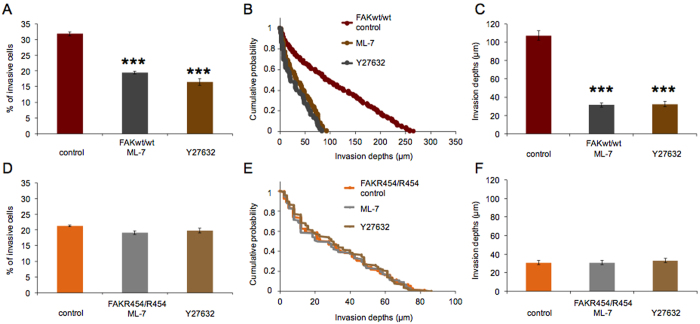
Inhibition of contractile forces using inhibitors of myosin light chain kinase (MLCK, ML-7) or Rho kinase (ROCK, Y27632) strongly reduces cell invasion of FAK^wt/wt^ cells. (**A**) The percentage (mean ± SD) of invasive cells is decreased after ML-7 or Y27632 treatment, (**B**) the invasion profiles are changed and (**C**) the average invasion depths are greatly decreased relative to vehicle-treated cells. By contrast, both the MLCK and the ROCK inhibitor have no effect on FAK^R454/R454^ cells as (**D**) the percentage (mean ± SD) of invasive cells is not altered, (**E**) the invasion profiles and (**F**) the invasion depths are not changed compared to vehicle treatment. Thus, residual invasiveness of FAK^R454/R454^ cells does not depend on actomyosin contractility-based forces. (n = 4, p*** < 0.001).

In order to determine the contribution of contraction forces to the cell invasiveness of FAK^wt/wt^ cells, we performed the 3D extracellular matrix invasion assay in the presence and absence of the myosin light chain kinase (MLCK) inhibitor ML-7 (15 μM) and the Rho kinase (ROCK) inhibitor Y27632 (100 μM), respectively. As expected, both inhibitor treatments reduced the invasiveness of wild-type fibroblasts relative to vehicle treatment, by decreasing the percentage of invasive cells and by reducing the average invasion depths to one third, from 107.2 ± 5.2 μm (n = 238) observed for vehicle-treated controls to 31.3 ± 2.1 μm (n = 148) for ML-7 and 32.3 ± 2.8 μm (n = 91) for Y27632-treated cells, respectively (Fig. 8A–C). These results confirm that actomyosin-based contractility makes a critical contribution to cell migration into 3D extracellular matrices. Finally, the matrix invasion of FAK^R454/R454^ cells was analyzed in the presence and absence of MLCK or ROCK inhibitors to investigate whether residual invasiveness that can be observed for cells lacking FAK kinase activity, still depends on actomyosin contractility. However, invasiveness of FAK^R454/R454^ cells is not altered after treatment with either inhibitor, ML-7 or Y27632, affecting neither the number of invasive cells nor their invasion profiles or depths (Fig. 8D–F). The average invasion depths were 30.9 ± 2.1 μm (n = 127) for vehicle-treated FAK^R454/R454^ cells, 30.7 ± 2.3 μm (n = 112) and 33.0 ± 2.2 μm (n = 115) after ML-7 and Y27632 treatment, respectively (Fig. 8F). Consistent with our observations for cell adhesion and stiffening, testing for integrin-binding, these results for inhibitors of contraction forces indicate that cells lacking FAK kinase activity cannot transmit actomyosin contractility for the invasion of 3D confined matrices. Affecting the same pathway, control of FAK function may also require myosin II activity.

## Discussion

The invasiveness of cells is regulated by their biochemical and mechanical properties^48^,^55^. Besides adhesion/de-adhesion, cellular stiffness, cytoskeletal remodeling, extracellular matrix degradation by metalloproteinases^56^, generation and transmission of contractile forces play a prominent role in the speed of cellular invasion into 3D extracellular matrices^45^,^55^. As the transmission of contractile forces depends on the adhesion of cells to the extracellular matrix through focal adhesions, cytoskeletal adaptors such as talin, vinculin, paxillin, under the control of signaling proteins in particular FAK and Src are required to facilitate the coupling and, subsequently, provide the connection between the actomyosin cytoskeleton and the surrounding microenvironment^7^,^57^.

It is known that the cytoplasmic tyrosine kinase FAK regulates the dynamics of integrin-based cell adhesions during assembly and disassembly through different mechanisms^22^,^58^. Until now, FAK’s function in the migration process is documented mainly based on 2D motility assays^22^. Thus, the impact of FAK expression (and function) on the biomechanical properties of cells and transmission of forces has been proposed but not studied in detail. Analyzing cell motility in dense 3D collagen matrices, we find that the FAK kinase activity plays a central regulatory role in contractility-based cell invasiveness by providing strong adhesions.

Firstly, loss of FAK (and Pyk2) protein expression strongly inhibits cell motility into dense 3D extracellular matrices. In contrast to a study showing that Pyk2 compensates FAK loss in 2D migration assays on fibronectin-coated substrates^28^, siPyk2-silencing in FAK^wt/wt^ cells led to a 2/3 reduction of FAK and Pyk2 and reduced 3D collagen matrices invasion to similar levels as knock-down of FAK. Furthermore, in FAK^−/−^ fibroblasts no compensating effect of Pyk2 overexpression was observed on 3D invasion of cells. The main difference between 2D migration^28^ and 3D migration is that the motility assays performed are not comparable, as, for 3D invasion into dense collagen fiber matrices mechanical properties such as cellular stiffness, contractile forces and matrix remodeling play a role. Thus, in cell invasion, FAK signaling appears to function differently to Pyk2 and even up-regulation of Pyk2, reported to partially compensate loss of FAK function^51^, does not further increase the impaired invasiveness after loss of FAK. Furthermore, the requirement for FAK protein function is confirmed also by siFAK-silencing of human MDA-MB-231 breast cancer cells leading to a sharp drop in cell invasive behavior.

Secondly, expression of the R454 FAK mutant protein, lacking ATP-binding and kinase activity, does not support cell invasiveness, suggesting a central role for FAK kinase activity. Since FAK function is involved in multiple cellular processes, it is important to note that FAK^R454/R454^ cells, other than FAK^−/−^ cells, do not have problems with cell proliferation and spreading but show enhanced formation of focal adhesions and defects in 2D cell migration with respect to polarity and directional persistence^11^. Furthermore, in a forced 3D cell migration assay, in which cells are grown on a 2D substrate and embedded with a non-polymerized collagen gel solution on top of the cells, FAK knockdown impaired cell motility^59^. The forced 3D motility assay, however, is not comparable to the 3D invasion assay used in our study. In the invasion assay, cells are cultured on top of a polymerized collagen matrix and invade individually in the z-direction (directed motion) where the medium is still fresh and less cells are present.

In the absence of FAK kinase activity, adhesion coupling and control of the actin cytoskeleton, as well as cellular motility appear to be altered. Therefore, it is worth to analyze signaling defects, which may cause the severe effect on invasiveness of FAK^R454/R454^ cells not anticipated by their characteristics in 2D migration. FAK kinase activity regulates the activation of Src^18^, which has been demonstrated to enhance cell invasion in human lung carcinoma cells^60^,^61^,^62^,^63^. Moreover, a downstream signaling molecule, p130CAS, has been reported to be involved in cell motility/invasion^64^, and indeed, p130CAS^−/−^ cells show decreased transmission of contractile forces compared to wild-type cells^65^.

To evaluate which factors may contribute to the behavior of FAK^R454/R454^ cells, the model of FAK signaling in adhesion sites should be considered more closely. Canonical FAK signaling models emphasize the role of FAK Tyr-397 (auto)-phosphorylation creating a binding site for Src family kinases, which leads to the formation of an active multiprotein FAK-Src signaling complex^16^ and subsequently to the phosphorylation of focal adhesion proteins, such as p130Cas, paxillin and vinculin, controlling adhesion site dynamics^7^. FAK^R454/R454^ cells were established from E8.5 FAK^R454/R454^ embryos, and both, cells as well as embryos do not show FAK kinase activity or phosphorylation of Tyr-397^11^, suggesting impaired mechanical coupling during cell adhesion. Although there is no canonical site for Src docking and activation in the adhesion process, FAK^R454/R454^ embryos and cells show normal to elevated tyrosine phosphorylation of paxillin and p130Cas. Moreover, in FAK^R454/R454^ cells, even Tyr-416 phosphorylation of Src is enhanced^11^, revealing that Src activity and tyrosine phosphorylation of focal adhesion proteins are not blocked in cells lacking FAK kinase activity (and Tyr-397 phosphorylation). Consistent with enhanced Src activity, we observed higher cellular stiffness and decreased deformability in suspended FAK^R454/R454^ cells, as shown by atomic force microscopy and optical cell stretcher experiments. However, adherent FAK^R454/R454^ cells are softer than adherent FAK^wt/wt^ cells and show a reduced stiffening response to matrix adhesion. These results confirm the general model of FAK signaling, whereby formation of the FAK-Src signaling complex, which requires FAK kinase activity (Tyr-397 phosphorylation), is central to adhesion dependent mechano-coupling and transmission of contractile forces.

Thirdly, we find that FAK kinase activity is required for cellular control of stiffness and adhesion strength. Both parameters are expected to critically affect the adhesion-based invasiveness of cells. Whether cells in general need to be softer or stiffer to invade dense 3D extracellular matrices is still a controversy and may depend on which state, such as adhesion or suspension, cell stiffness is measured and which cell types, such as fibroblasts, mesenchymal cells, epithelial cells, specific cancer cell types or amoeboid cells, are analyzed^12^,^47^,^66^,^67^. In line with our previous reports, the stiffer fibroblasts are more invasive than softer fibroblasts^9^,^37^,^67^. In FAK^R454/R454^ cells, the cellular stiffness is significantly reduced, which is consistent with a previous report showing that FAK^−/−^ cells are softer^68^, while detachment of fibronectin-coated and collagen-coated beads is enhanced, indicating that the adhesion strength is reduced in FAK^R454/R454^ cells. This result seems to be in contrast to previous reports showing that the adhesion strength of FAK^−/−^ cells (not FAK^R454/R454^ cells), which was determined by measuring shear stress using a spinning disc device, is increased compared to FAK^wt/wt^ cells^69^,^70^. Although there were different methods applied to determine adhesion forces, and furthermore, FAK^−/−^ cells show a different adhesion and spreading behavior than FAK^R454/R454^ cells, we cannot fully resolve the reason for these conflicting data. In our hands, FAK^R454/R454^ cells behave consistently in all assays, and using atomic force microscopy, we collected further evidence that the adhesion strength of FAK^R454/R454^ cells, which are deficient for FAK kinase activity only, is lower than that of FAK^wt/wt^ cells. In addition, FAK^R454/R454^ cells show reduced stress-stiffening behavior compared to FAK^wt/wt^ cells, suggesting that FAK activity contributes to mechanosensing of adhesion site similar to what has been reported for integrins using magnetic twisting cytometry^32^. Consistently, in MDA-MB-231 cell migration, vinculin-related strengthening of adhesion sites was linked to activation of FAK signaling^52^. Furthermore, cells expressing the R454 FAK mutant show a very elongated phenotype in dense 3D matrices, and flow field analysis around invaded cells reveals impaired force transmission towards the collagen matrix. Thus, FAK^R454/R454^ cells appear to be defective in the transmission of contraction and compressive forces. This is also supported by atomic force measurements showing that adherent FAK^R454/R454^ cells exert lower forces compared to adherent FAK^wt/wt^ cells. These observations apparently contradict the increase in adhesion area that was reported for FAK^R454/R454^ cells^11^, however, in these reports the adhesion strength was not measured directly. Furthermore, the morphology of adhesion sites displayed by these cells in 2D adhesion is fuzzy and resembles immature, weak focal adhesions. In contrast, FAK^wt/wt^ cells show distinct, sharply outlined mature adhesion sites^11^. To what extent the induction of Pyk2 phosphorylation observed in FAK^R454/R454^ cells can affect biomechanical parameters remains to be established. Observations in FAK^−/−^ cells suggest an effect of Pyk2- up-regulation on the generation and transmission of contractile forces^68^,^71^. However, these studies tested the biomechanical properties of cells lacking all aspects of FAK protein function, including a defect in cell spreading i.e. control of actin dynamics.

Finally, residual invasiveness of cells lacking FAK kinase activity is not sensitive to inhibitors of Erk and Src signaling, whereas FAK^wt/wt^ cells lose 70% of their invasion depth, if these pathways controlling integrin adhesion and contraction-force based motility^22^ are inhibited. Moreover, FAK^R454/R454^ cells apparently cannot transmit contraction forces, since inhibition of myosin II-dependent contractility using inhibitors of ROCK or MLCK, does not alter invasiveness in these cells. These results clearly indicate that FAK kinase activity is required to control mechanical coupling of contraction force-mediated invasion. Its loss reduces cell invasiveness in 3D matrices to motility based on actin dynamics, as described for cells entirely lacking expression of integrin receptors^72^.

Which defects may cause the observed loss of adhesion and actomyosin contractility- based motility in cells without FAK kinase activity? Adhesion assays performed with FAK^R454/R454^ cells indicate a defect in the control of Rho A and Rac activities, leading to de-regulated, high activation of both small G proteins^11^, however, their spatiotemporal control is a critical requirement for the coordination of actin dynamics and force coupling in adhesion sites^7^. In particular, p190Rho GAP was shown to require FAK kinase activity for its localization and subsequent local signaling to reduce activation of RhoA^73^. Therefore, de-regulation of RhoA and Rac activities in FAK kinase deficient cells and tissues (FAK^R454/R454^) appear to uncouple Src/Erk signaling from generation and transmission of forces in focal adhesions and lead to loss of actomyosin-based cell invasiveness. Consistently, observations in transgenic animals emphasize the importance of FAK kinase activity. Loss of the FAK Tyr-397 auto-phosphorylation and docking site in Δexon15 FAK mice, leads to a semi-penetrant lethality at E14.5 with some viable offspring^74^, while FAK^R454/R454^ mutant embryos all die around E8.5^11^, coinciding with lethality of the vinculin knock-out and critical force-dependent steps in embryonic development of heart and brain (neural tube closure)^67^,^75^.

Taken together, this study reveals that the active kinase FAK plays a role in facilitating fibroblast and cancer cell invasion in artificial dense 3D extracellular matrices (with an average pore-size of 1.3 μm). Furthermore, it points out that the active kinase domain of FAK is necessary to initiate and promote the invasiveness of cells based on the spatiotemporal control of the FAK-Src signaling complex in adhesion sites. The ability to transmit and generate contractile forces through ROCK kinase and MLCK is necessary to facilitate FAK-dependent cell invasion. These findings indicate that the focal adhesion protein FAK plays a central role as a mechano-coupling and mechano-regulating protein, promoting 3D motility through regulation of cell mechanical properties.

## Experimental Procedures

### Cells and cell culture

Mouse embryonic wild-type fibroblasts (FAK^wt/wt^) as well as FAK deficient fibroblasts (FAK^−/−^) were purchased from ATCC-LGC-Promochem (Wesel, Germany) and cultured as described for the fibroblasts above. We used accutase to harvest the cells and obtained below 1% dead cells. The FAK^R454/R454^ cells were kindly provided by Dr. Schlaepfer. In brief, the R454 FAK knock-in point mutation (lysine K454 to arginine R454) was generated by homologous recombination (InGenious Targeting Laboratory, Stony Brook, NY) as described^11^. Primary FAK^R454/R454^ (kinase-dead) and FAK^WT/WT^ mouse embryonic fibroblasts (MEFs) were isolated from E8.5 embryos (of a mixed C57BL/6 129/SvEv background)^11^. Immortalization of primary MEFs were performed using retrovirus-mediated expression of human telomerase reverse transcriptase (hTERT) (Addgene, Cambridge, MA) and selected for puromycin. Fibroblasts were maintained in high glucose (4.5 g/liter) Dulbecco’s modified Eagle’s medium supplemented with 10% fetal calf serum (with a low endotoxin level < 0.1 EU/ml), 2 mM L-glutamine, and 100 units/ml penicillin/streptomycin (DMEM complete medium; all from Biochrom, Berlin, Germany). The human breast carcinoma cell line MDA-MB-231 was purchased from ATCC-LGC-Promochem and was maintained in low-glucose (1 g/l) DMEM supplemented with 10% FCS (low endotoxin, <0.1 EU/ml), 2 mM L-glutamine and 100U/ml penicillin–streptomycin (Biochrom, Berlin, Germany). 80%-confluent cells were used in passages 6–40. We bought all chemicals and drugs from Sigma (Taufkirchen, Germany) unless otherwise indicated.

### 3D extracellular matrix invasion assay

3D collagen invasion assays were utilized to follow invasion of fibroblasts or human breast cancer cells over hours to several days. For a 6-well plate a mixture of 3.5 ml collagen R (Serva, Heidelberg, Germany), 3.5 ml collagen G (Biochrom, Berlin, Germany), 0.8 ml of 278 mM sodium bicarbonate (end concentration 26.5 mM) and 0.8 ml 10X DMEM (Biochrom) was prepared by avoiding air bubbles during the entire process. The polymerization starts directed after neutralization of the collagen mixtures with 1 N sodium hydroxide. In each well of the plate, 1.2 ml collagen mixture was given and gels were polymerized at 37 °C, 5% CO_2_ and 95% humidity to 500 μm thick collagen fiber matrices for at least two hours. These 3D extracellular matrices were incubated overnight with 2 ml DMEM^47^. 1*10^5^ cells were seeded on top of the 3D extracellular matrices and incubated for 72 h at 37 °C, 5% CO_2_ and 95% humidity in DMEM with 10% FCS. After three days, alterations in the migratory capacity of the cells were clearly observable. After the fixation with 2.5% glutaraldehyde solution buffered with PBS, the percentage of invasive cells and their invasion depths were analyzed in 12 randomly-selected fields of view. The percentage of invasive cells (cells inside 3D extracellular matrices) was determined from total cells, which means adhesive cells on the surface of the 3D extracellular matrices and invasive cells inside the 3D extracellular matrices^47^.

### Flow field analysis

FAK^wt/wt^ and FAK^R454/R454^ cells were seeded on 3D collagen matrices and incubated for three days in a cell incubator. The invaded cells in the 3D matrix were placed under a microscope with an environmental chamber enabling life cell imaging (Zeiss, Germany) and analyzed for at least 1.5 up to 3 hours by taking phase contrast images of single cells in a gel every five minutes. For the determination of the flow field we used the Lucas-Kanade Algorithm^76^, and implemented it into a self-written program (Python). In particular, the flow was analyzed in twenty scrapings around the cell with each of 1.9 μm thickness.

### Modulation of cell invasion

In order to inhibit or alter the invasion of the cells, we added 15 μM myosin light chain kinase inhibitor ML-7 (Calbiochem) and 100 μM Rho-kinase inhibitor Y27632 (Sigma) to the 3D extracellular matrix invasion assay prior to cell seeding and cultured the cells for three days. Furthermore, to analyze the FAK-mediating signaling, we inhibited or altered cell invasion by addition of 30 μM a Src tyrosine kinase inhibitor, termed ‘4-(4′-phenoxyanilino)-6,7-dimethoxy-qinazoline’ (Cat. No. 567805, Calbiochem) or 10–20 μM Erk-inhibitor called ‘ERK activation inhibitor peptide II’ (Cat. No. 328005, Calbiochem).

### siRNA Transfection

80%-confluent cells were harvested and 200,000 cells were seeded in Ø 3.5 cm dishes and cultured in 2 ml DMEM with 10% FCS. 5 μl of a 20 μM FAK (target sequences for FAK: murine siFAK GGGCAUCAUUCAGAAGAUA^77^ and human siFAK GGTTCAAGCTGGATTATTT^78^, Pyk2 (target sequence for Pyk2: siPyk2 GGGACAUUGCUGCUCGGAA)^71^ or Allstar-control RNAi-solution (control-siRNA), 12 μl HiPerFect-Reagent (Qiagen) and 100 μl DMEM were mixed and cells transfected according to manufacturer’s protocols^47^.

### mRNA-isolation and realtime PCR for mRNA quantification

RNA isolation, reverse transcription, and realtime PCR were performed using the RNeasy Mini Kit, the Reverse Transcriptase Kit, and the Quantifast SYBR Green Kit all from Qiagen (Hilden, Germany), according to manufacturer’s protocols. At indicated time points after transfection, cells were washed twice with PBS (37 °C) and lysed directly into RLT buffer. Quantitative PCR was performed on the 7900 HT Fast Realtime PCR System (Applied Biosystems, Darmstadt, Germany) using primers from Qiagen for detection of the following genes (human/murine); ***PTK2/Ptk2***(FAK): Hs_PTK2 PPH02827A/Mm_Ptk2 PPM35305B (RT^2^ qPCR Primer Assay); ***Ptk2**b*(Pyk2): Mm_Ptk2b PPM04918B (RT^2^ qPCR Primer Assay); ***HPRT/Hprt***: Hs HPRT fw (5′-3′) GCT GAC CTG CTG GAT TAC, Hs HPRT rv (5′-3′) TGC GAC CTT GAC CAT CTT, Mm_Hprt_1_SG, QT00166768 (QuantiTect Primer Assay), ***B2M/B2m***: Hs_B2M_1_SG, QT00088935 (QuantiTect Primer Assay), Mm B2m fw (5′-3′) GGA CTG GTC TTT CTA TAT CCT GGC, Mm B2m rv (5′-3′) GTC TCG ATC CCA GTA GAC GG, ***GAPDH/ Gapdh***: Hs_GAPDH_2_SG, QT01192646 (QuantiTect Primer Assay), Mm Gapdh fw (5′-3′) TAT GTC GTG GAG TCT ACT GG, Mm Gapdh rv (5′-3′) AGT GAT GGC ATG GAC TGT GG and mRNA levels of *PKT2/Ptk2/Ptk2b* determined using three reference genes^79^.

### Fluorescence-based immunoblot quantification of protein expression

Protein levels of FAK and Pyk2 were determined from whole cell extracts using β-tubulin and α-actinin as reference to control the loading of each line. All probes were analyzed at least twice and similar results were obtained in independent repeat experiments. At indicated time points after transfection, cells were washed twice with PBS (37 °C) and lysed directly into 2x Laemmli buffer (6x Laemmli; 50 mM Tris base, 5 mM EDTA, 5% SDS, 0.2 M DTT, 50% glycerol, bromophenol blue, pH 8.0). For each cell line, whole cell lysates of different treatment conditions siControl/siFAK (siPyk2) were loaded in parallel for all days (d0, d1, d2, d4) and analyzed by immunoblotting using fluorescently labeled secondary antibodies. Quantitative fluorescence detection was performed on the Odyssey Fc System (Li-Cor Biosciences, Bad Homburg, Germany) using ImageStudio 5.2 (Li-Cor Biosciences) to determine band intensities. Primary antisera used were: pc rabbit anti FAK (C-20) (SC-558, Santa Cruz Biotechnology, Heidelberg, Germany), mc mouse anti FAK2 (5E2D5) (Life Technologies, Darmstadt, Germany), pc rabbit anti β-tubulin (SC-9104, Santa Cruz Biotechnology) and mc mouse anti α-actinin (BM-75.2) (Sigma Aldrich, Taufkirchen, Germany) and secondary antisera, IRDye-800CW Goat anti-Rabbit IgG, polyclonal and IRDye-800CW/680RD Goat anti-Mouse IgG, polyclonal (Li-Cor Biosciences).

### Imaging within 3D extracellular matrices using confocal laser scanning microscopy

Glass cover slips are activated with plasma-cleaner for 5 min, silanized in a spin-coater with (3-aminopropyl) triethyoxysilane (Sigma Aldrich), then washed 2–3 times with dH_2_O, incubated with 2.5% glutaraldehyde at 37 °C, washed with dH_2_O. Then the glass is coated with collagen type I for 2 hours at 37 °C, washed and incubated with complete medium for 24 hours. Between 3000 and 5000 cells were seeded on 3D extracellular matrices mounted on a cover slip (for gel preparation see invasion assays) and cultured for two days. The cells were fixed with 2.5% glutaraldehyde, washed at least twice with a PBS-buffer, permeabilized with 0.1% Triton-X100 for 5 min at room temperature, washed and incubated overnight with 1% BSA Hepes-buffer. Cells were stained with 10 units/ml Alexa Fluor 546 at 4 °C dissolved in 1% BSA Hepes-buffer for 2.5 hours. After rinsing with 1% BSA Hepes-buffer, the glass cover slips were treated with prolong gold antifade and mounted on a glass slide. After 24 hours at 4 °C, when antifade acquired a gel-like consistency, chambers were sealed with laquer and analyzed directly under the laser scanning microscope (Leica TCS SP2, Wetzlar, Germany). The cell’s actin network was analyzed in terms of stress fiber thickness in a 3D extracellular matrix and the migration speed as well as the persistence of migration through 3D extracellular matrices (time-lapsed videos over at least 1.5 up to 20 hours) were analyzed using image J software.

### Magnetic Tweezers

Using magnetic-tweezer microrheology, a staircase-like sequential pattern of forces (0.5–10 nN) were applied to 4.5 μm beads (superparamagnetic and epoxylated) coated with 100 μg/ml fibronectin from human plasma (Roche Diagnostics, Mannheim, Germany, Cat. No. 11080938001) or 100 μg/ml bovine collagen type I^47^,^80^. 2*10^5^ sonificated beads were added to 10^5^ cells, and incubated for 30 min at 37 °C, 5% CO_2_ and 95% humidity. Measurements were performed at 37 °C using an inverted microscope (DMI-Leica). After that time, the beads are strongly bound to the cell’s cytoskeleton, and molecular details of the bond seem to be negligible and thus have no influence on the bead motion^81^. The cells showed a creep response J(t) during force application, which can be described by a power-law in time, J(t) = a(t/t_0_)^b^ with a and exponent b depending on the force, and a reference time t_0_ being set to 1 s. The displacement of the beads in response to increasing staircase-like forces followed a superposition of power-laws^82^ from we determined the force-dependence of a and b by using a least-squares fit^47^. The factor a is given in units of μm/nN and describes the elasticity of the cells and corresponds to the compliance (being the opposite stiffness)^47^. In particular, the force/distance relationship (in units of nN/μm) can be related to cellular stiffness (in units of Pa) by a geometric factor, which is regulated by bead/cell contact area and the entire cell height. Through knowledge of those parameters, this geometric factor is estimated using finite element analysis^83^. Without knowledge of cell’s height and degree of bead internalization, a typical strain ε is estimated by dividing bead displacement d by bead radius r, and a typical stress ε by dividing the exerted force F by bead cross-sectional area πr^2^ resulting in a formula for the cellular stiffness: G = σ/ε = r/d ∙F/(πr^2^)^84^. For 4.5 μm bead, a geometric factor has the value 0.14 μm^−1^. Moreover, a cell with an apparent stiffness of 1 nN/μm would be stiffness of 140 Pa stiff.

The exponent b represents the bond-stability of force-bearing cellular structures bound to the coated beads. An exponent b of a value 1 means it behaves as a Newtonian-viscous liquid and a value of 0 as an elastic solid^85^,^86^. As “cellular” b values are between 0 and 1, it means that the deformation energy is not fully elastically stored in the cell’s cytoskeleton, and has dissipated as heat due to the remodeling of the cell’s cytoskeleton to which the bead is coupled^87^. Hence, dissipation is coupled to the remodeling events breaking these elastic bonds and their turn-over. The turn-over of these acto-myosin structures accounts for the dissipative properties^88^ and hence for contractility-driven shape changes in the cell’s cytoskeleton.

In cells, the exponent b usually has values between 0.1 and 0.5 with values close to 0.5 indicating high turn-over rates of cytoskeletal structures^85^,^89^. Both, a and b, values are averaged over all exerted forces for all beads and are presented as mean ± SE.

### Scanning electron microscopy

Adherent cells with bound fibronectin or collagen type I-coated (see above Magnetic Tweezers) are fixed with 2.5% glutaraldehyde-fixed cells, dehydrated with an ethanol series, washed with hexadimethylsilazane reagent (Electron-Microscopy-Science, Hatfield, PA) and air-dried^45^,^47^. After sputter-coating with gold, FAK^wt/wt^ and FAK^R454/R454^ cells were analyzed using scanning electron microscopy (SEM) (ISI-SX-40, International Scientific Instruments, Milpitas, CA) (n = 3 repeat experiments).

### Atomic force microscopy

The cantilevers were coated with 20 μg/ml fibronectin over night at 4 °C. Before inserting the cantilever for atomic force microscope measurements, the glass block was cleaned with double distilled endotoxin-free water ddwater (dH_2_O) and ethanol. The spring constant of both cantilevers was determined in dH_2_O of both cantilevers with thermal noise method (second peak was used). The cells were harvested using 0.125% Trypsin/EDTA for 5 min, seeded into plastic culture dishes, which were placed onto the heated stage (37 °C) with 5% CO_2_ influx. After 2 hours the cells were measured by pressing the cantilever on top of the cell for 30 s. The approach and retraction force-distances curves were recorded. The maximum force was determined by analyzing the data with the JKP software. In order to determine the Young’s modulus a 5.9 μm polystyrol bead was glued to the cantilever, which was pressed with 0.5 nN on the adherent cells. For measurement of the suspended cells (non-adherent cells) a flat cantilever was pressed on the cells with a force of 1.5 nN.

### Optical Cell stretcher

For each optical cell stretcher measurement, the FAK^wt/wt^ and FAKR^454/R454^ cells were cultured at 37 °C and 5% CO_2_ to 70% confluency in a T25 cell culture flask, were harvested in 3 min using a 0.125% trypsin/EDTA solution (PAA). Suspended cells were measured with an automated Optical Cell Stretcher setup by recording the cell deformation using a CCD video camera (Baseler, Switzerland) for image acquisition, as described^66^,^90^,^91^. A microfluidic pump system place on an inverted microscope transports single cells to the optical trap region. The optical stretcher consists of a dual beam laser trap (two single mode CW Ytterbium fiber lasers with a wavelength of 1064 nm) that traps (100 mW laser power) and deforms single cells through optically induced stress acting on the cell’s surface (at 1000 mW laser power). The flow chamber is connected to two co-axial optical fibers that are placed perpendicular to a square glass capillary containing cells. A cell was trapped between two opposite divergent laser beams^92^ for one s and the cell stretch occurred by increasing the laser power from 100 mW to 1000 mW for two seconds. Relaxation was measured by reducing laser power to 100 mW for two seconds. As a response to a laser-induced stretch along the cell’s long axis, the cell shows creep behavior.

Cellular deformation data analysis: A broad range of cells was measured. We excluded irregular-shaped cells, as they may introduce rotations during the cell stretch leading to ‘false’ (smaller or larger) deformations. Microfluidic flow was stopped before cell trapping occurred to minimize cellular rotations and wobbling. Air bubbles within the capillary and cell debris in suspension was omitted to avoid a non-uniform pressure gradient possibly disturbing microfluidic flow. The relative cell deformations were derived via an automated subpixel edge detection algorithm implemented in Matlab (MathWorks)^66^,^91^. This algorithm corrects small angle rotations of the trapped cell using feature tracking. All remaining cells are evaluated with respect to their creep deformation J(t) = ε(t)/ σ_0_, where ε(t) = [d(t)-d(0)]/d(0) is the relative deformation of a cell along the long cell axis (the laser axis), and σ_0_ is the optically induced stress depending linearly depends on the laser power^66^. The optical cell deformation followed a non-Gaussian distribution and thus, values were presented as medians. As the analysis form of the distributions for evaluation of the cellular mechanical properties is still under strong debate, the bootstrapping method was used to estimate the 95.46% confidence interval (2*SD). After generation of a median-bootstrapping distribution with 10000 iterations it was fitted with a Gaussian function to approximate standard deviation of the median.

### Statistical analysis

The data were expressed as mean values ± SD unless otherwise stated. For all statistical analyses a two-tailed Student’s t-test was performed. We considered a p-value below 0.05 as statistically significant.

## Additional Information

**How to cite this article:** Mierke, C. T. *et al*. Focal adhesion kinase activity is required for actomyosin contractility based invasion of cells into dense 3D matrices. *Sci. Rep.* **7**, 42780; doi: 10.1038/srep42780 (2017).

**Publisher's note:** Springer Nature remains neutral with regard to jurisdictional claims in published maps and institutional affiliations.

## Supplementary Material

Supplementary Information

Supplementary Video 1

Supplementary Video 2
