# Corrigendum: Focal adhesion kinase activity is required for actomyosin contractility-based invasion of cells into dense 3D matrices

**DOI:** 10.1038/srep46435

**Published:** 2017-05-03

**Authors:** Claudia T. Mierke, Tony Fischer, Stefanie Puder, Tom Kunschmann, Birga Soetje, Wolfgang H. Ziegler

Scientific Reports 7: Article number: 42780; 10.1038/srep42780 published online: 02 16 2017; updated: 05 03 2017.

This Article contains an error in the legend of Figure 5F.

“(**F**) The Young’s modulus of suspended FAK^wt/wt^ cells (blue, n = 20) is higher compared to suspended FAK^R454/R454^ cells (red, n = 20)”.

should read:

“(**F**) The Young’s modulus of suspended FAK^wt/wt^ cells (blue, n = 20) is lower compared to suspended FAK^R454/R454^ cells (red, n = 20)”.

