# Supplementary material for: Focal adhesion kinase activity is required for actomyosin contractility-based invasion of cells into dense 3D matrices
Source: Sci Rep. 2017 Feb 16;7:42780. doi: 10.1038/srep42780 (PMC5311912; doi:10.1038/srep42780)
Supplement: Supplementary Information [file srep42780-s1.pdf]

Supplementary Information

FOCAL ADHESION KINASE ACTIVITY IS REQUIRED FOR ACTOMYOSIN CONTRACTILITY BASED INVASION OF CELLS INTO DENSE 3D MATRICES

Claudia T. Mierke*[1], Tony Fischer[1], Stefanie Puder[1], Tom Kunschmann[1], Birga Soetje[2], and Wolfgang H. Ziegler[2]

[1]Institute of Experimental Physics I, Biological Physics Division, Faculty of Physics and Earth Science, University of Leipzig, Leipzig, Germany
[2]Department of Paediatric Kidney, Liver and Metabolic Diseases, Hannover Medical School, Hannover, Germany

*Address correspondence to: claudia.mierke@uni-leipzig.de

Supplementary Figure S1

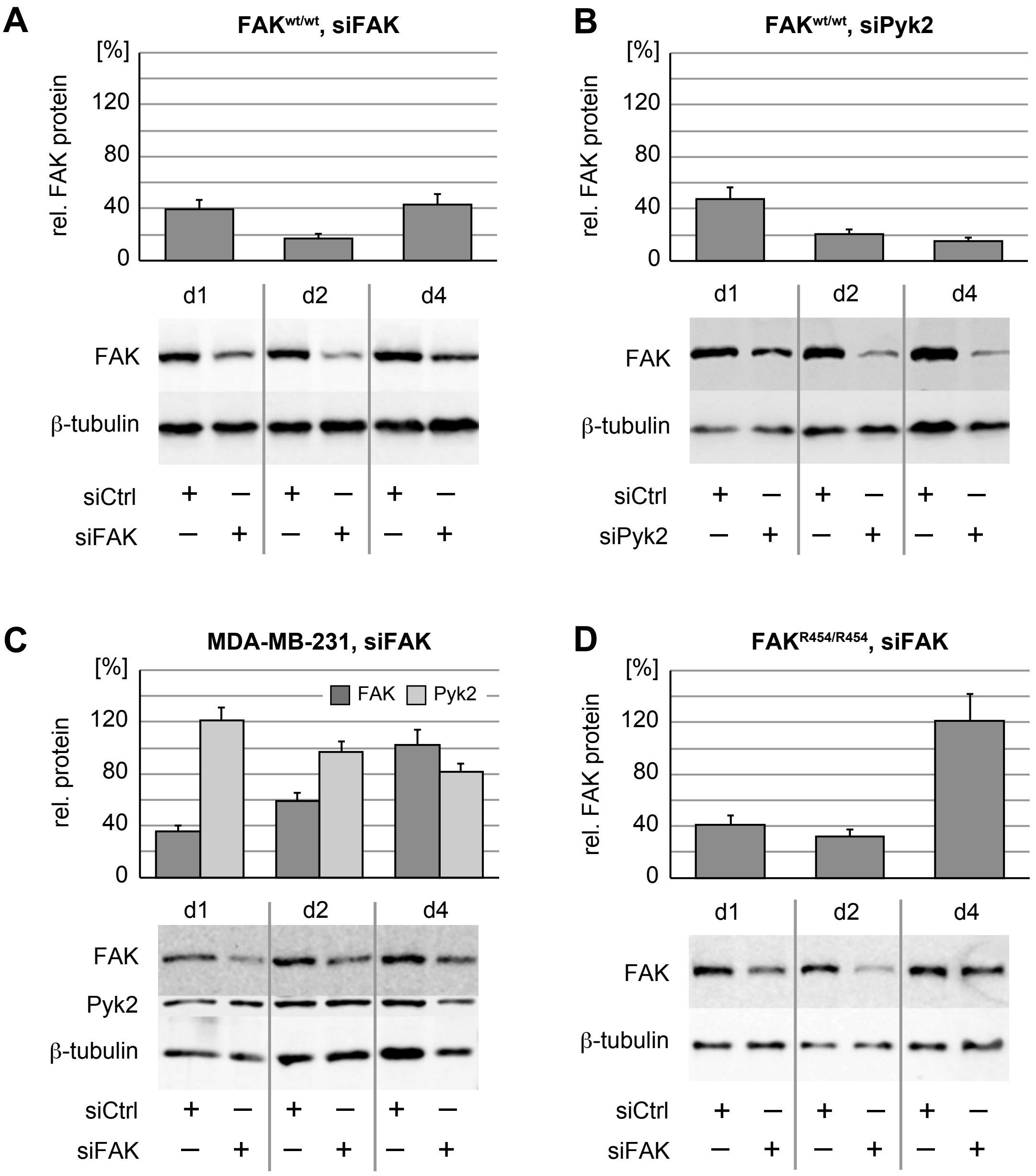

Figure S1: Fluorescence intensity-based quantification of FAK (and Pyk2) protein expression following siRNA-mediated knockdown. After 1- 4 days (d1, d2, d4) of treatment with siFAK (or siPyk2), protein levels were determined in different cell lines $FAK^{wt/wt}$, MDA-MB-231, and $FAK^{R454/R454}$ (A-D). Relative protein levels, mean ± SEM, were determined as signal ratios of siFAK(siPyk2) to siCtrl- treated cells for each day, after normalization to two reference proteins β-tubulin and α-actinin (total protein load) in respective lanes. All probes were analyzed at least twice. Signal variation was estimated based on normalized siCtrl signals (8 values / gel). Below graphs, representative protein bands of FAK (Pyk2; only MDA-MB-231) and β-tubulin of corresponding lanes are shown for siCtrl and siFAK(siPyk2)- treatment of cells, respectively.

Supplementary Table S1

	**FAK$^{wt/wt}$ (CRL-2645)**			
siRNA	siFAK		siPyk2	
mRNA quant.[1] [% control d0]	*Ptk2* [FAK]	*Ptk2b* [Pyk2]	*Ptk2* [FAK]	*Ptk2b* [Pyk2]
d1	53±5	176±71	36±3	44±18
d2	39±4	77±22	28±3	21±8
	**FAK$^{-/-}$ (CRL-2644)**			
	normalized to FAK$^{-/-}$		normalized to FAK$^{wt/wt}$	
siRNA	siPyk2		siPyk2	
mRNA quant.[1] [% control d0]	*Ptk2* [2] [FAK]	*Ptk2b* [Pyk2]	*Ptk2* [2,3] [FAK]	*Ptk2b* [3] [Pyk2]
d1	96±13	71±15	10±1	464±96
d2	75±10	51±11	8±1	334±69
	**MDA-MB-231**		**FAK$^{R454/R454}$**	
siRNA	siFAK		siFAK	
mRNA quant. [% control d0]	*Ptk2* [4] [FAK]	*Ptk2b* [Pyk2]	*PTK2* [1] [FAK]	*PTK2b* [Pyk2]
d1	22±1	(protein only)	27±3	87±2
d2	31±1		42±4	142±3

[1] mRNA quantification based on reference genes *Hprt, Gapdh, B2m* and normalization to controls, d0
[2] no full-length FAK protein detected, only 25 kDa C-terminal fragment (data not shown)
[3] mRNA quantification based on reference genes and normalization to d0 of MEF FAK$^{wt/wt}$
[4] mRNA quantification based on reference genes *HPRT, GAPDH, B2M* and normalization to controls, d0

Gene and protein names: *Ptk2/PTK2*, focal adhesion kinase; *Ptk2b/PTK2b*, Pyk2 / focal adhesion kinase 2

Supplementary Figure S2

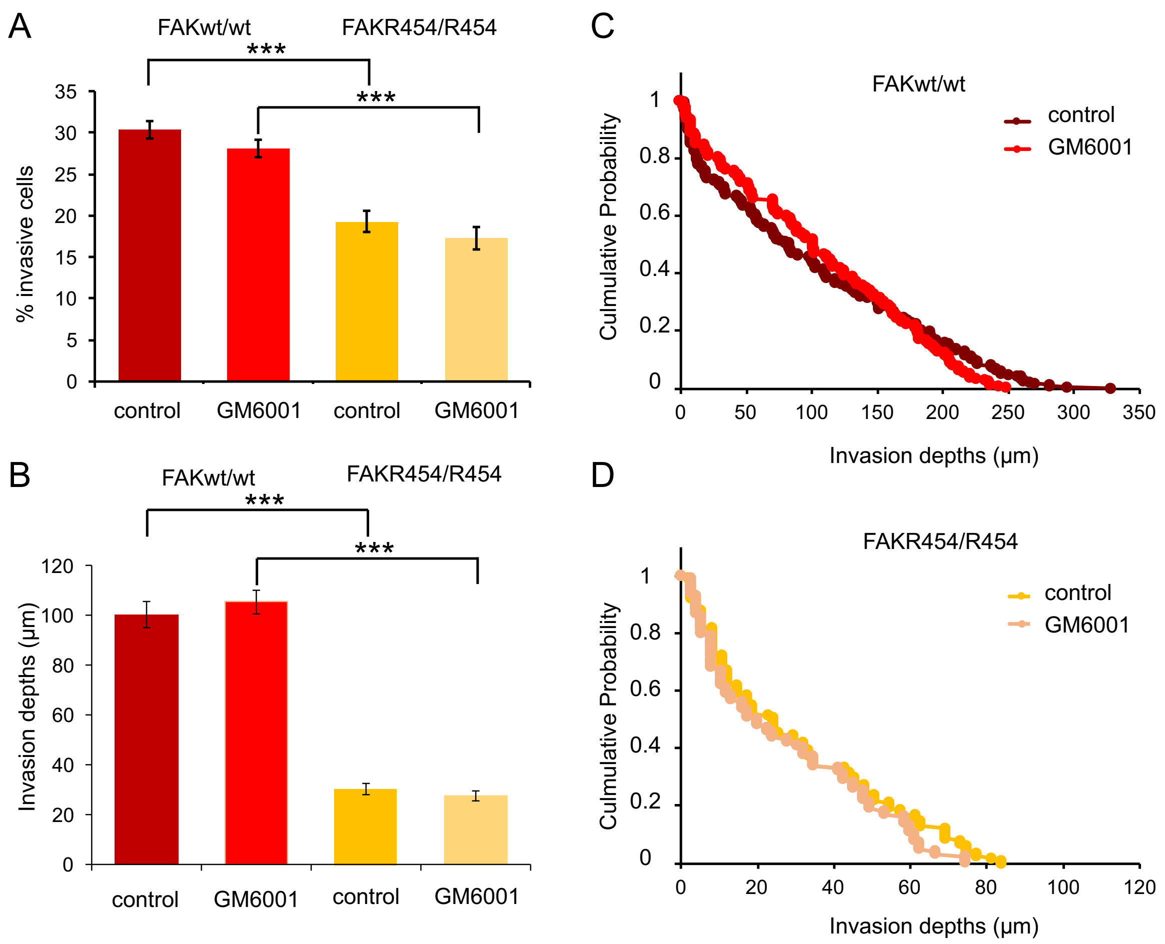

Figure S2: Invasion of $FAK^{wt/wt}$ and $FAKR^{454/R454}$ into 3D extracellular matrices for three days in absence or presence of the broad-range matrix-metalloproteinase inhibitor GM6001 (50 µM). (A) The percentage (mean ± SD) of invasive cells and (B) the invasion depths are significantly higher in $FAK^{wt/wt}$ cells (red) compared to $FAK^{R454/R454}$ cells (orange), when comparing both cell types under vehicle-treatment (dark colors) and 50 µM GM6001-treatment (light colors). (C) Invasion profiles of $FAK^{wt/wt}$ cells and (D) $FAK^{R454/R454}$ cells are not altered by GM6001 treatment compared to vehicle controls, but still significant different when comparing both cell types at either vehicle-treatment (dark colors) or GM6001-treatment (light colors). The experiment was repeated three times independently (***$p < 0.001$).

Supplementary video S1: A representative FAKwt/wt cell is migrating in a 3D collagen fiber matrix and alters the collagen network by pulling strongly on it (life cell phase contrast images).

Supplementary video S2: A representative FAKR454/R454 cell is migrating in a 3D collagen fiber matrix and alters the collagen network less (life cell phase contrast images).
